# Prevalence of Aflatoxin Contamination in Peanuts and Peanut Butter from an Informal Market, Harare, Zimbabwe

**DOI:** 10.1155/2022/3761078

**Published:** 2022-09-13

**Authors:** V. P. Masaka, N. Ndlovu, R. S. Tshalibe, T. C. Mhande, T. Z. Jombo

**Affiliations:** ^1^Midlands State University, Department of Food Science and Nutrition, P. Bag, 9055 Gweru, Zimbabwe; ^2^Central Veterinary Laboratory, 18A Borrowdale Road, P.O Box CY551, Causeway, Harare, Zimbabwe

## Abstract

Peanuts and peanut butter play an important role nutritionally in improving the diets of individuals in many parts of Africa, especially in the fight against child malnutrition. However, in developing countries such as Zimbabwe, most of the raw peanuts and peanut butter produced in backyard industries are sold in informal markets and rarely undergo formal safety inspection for aflatoxin contamination. The objective of the study was to determine the prevalence of aflatoxins in raw peanuts and backyard peanut butter sold at Mbare informal market. Ten (10) raw peanut samples and twenty (20) peanut butter samples were collected from Mbare informal market. Aflatoxin contamination was determined using liquid chromatography-mass spectrometry (LC-MS). The results revealed that sixty percent (60%) of the raw peanut samples were contaminated with total aflatoxin ranging from <0.75 to 426.4 *μ*g/kg. One hundred percent (100%) of peanut butter samples were contaminated with total aflatoxins ranging from 4.7 *μ*g/kg to 435.0 *μ*g/kg. Aflatoxin B_1_ was the most prevalent aflatoxin in both raw peanuts (range, 1.2 *μ*g/kg to 90.8 *μ*g/kg) and peanut butter (range, 4.7 to 382.9 *μ*g/kg). Forty percent (40%) of the raw peanuts and 95% of peanut butter samples exceeded the maximum limits of AFB_1_ as set by Zimbabwe legislation. The results suggest that raw peanuts and especially the peanut butter from backyard industries are heavily contaminated with aflatoxins and could constitute a possible health risk to consumers who regularly purchase these food commodities from informal markets.

## 1. Introduction

Peanut (*Arachis hypogaea* L.) is an important grain legume nutritionally since it is a good source of protein, dietary fibre, polyunsaturated fats, complex carbohydrates, and essential minerals [1, 2]. The legume plays a major role in the diet of infants and young children and is often consumed raw, boiled, roasted, or processed into peanut butter in most sub-Saharan African households [3]. Peanut butter is used as a spread on bread and for making sauces used in vegetable and meat dishes as well as adding to rice and samp [4]. However, the legume is highly susceptible to aflatoxin contamination during preharvest and postharvest periods posing a threat to food safety and health [5]. Aflatoxins are secondary toxic metabolites produced by the fungal genus Aspergillus and there are four major types, namely, Aflatoxin B_1_ (AFB_1_), Aflatoxin B_2_, Aflatoxin G_1_, and Aflatoxin G_2_ [6]. Aflatoxin contamination of peanuts occurs and increases at all stages involved in the supply chain which include the field, drying, and storage as well as the peanut based products [7, 8].

Aflatoxins' presence in agricultural produce is a food safety problem globally, especially in low-income countries because of their demonstrated toxicological effects in humans [9]. Epidemiological studies have shown AFB_1_ to be the most potent liver carcinogen amongst the four toxin types and are classified as a class 1 human carcinogen [10]. Furthermore, aflatoxins have been associated with immunotoxic effects [11], which have been blamed for the increased progression of HIV to AIDS in low-income countries. This is based on previous studies that have indicated a relationship between a high viral load and high levels of aflatoxins in Ghana, especially AFB_1_ [12]. Also, several studies which were conducted in Nigeria [13], Cameroon [14], and Egypt [15] established a link between chronic exposure of children to aflatoxins and the development of kwashiorkor, a severe Protein Energy Malnutrition (PEM) disease.

Adverse health effects from aflatoxins are of major concern especially in African countries due to high poverty rates and informalized economies leading to producers buying their groundnuts directly from farmers [16]. In Zimbabwe, approximately 75 percent of the peanuts are grown by smallholder farmers [17]; however, due to weak marketing arrangements, the grain ends up being traded more on the informal market. This market is a readily and reliable source of peanuts for the backyard industries. The peanut butter produced from such industries is normally sold in underregulated markets and at relatively lower prices than its commercial alternatives. The presence of aflatoxin has been previously reported in peanuts and peanut butter from formal and informal sectors in Bulawayo, Zimbabwe's second largest city [18]. However, there is still a need to further understand the levels of aflatoxin especially in informal markets in Zimbabwe. Therefore, the objective of the current study was to assess the prevalence of aflatoxin contamination in raw peanuts and peanut butter produced in backyard industries sold at the Mbare musika market, which is the largest informal market in Harare, the capital city of Zimbabwe. The data will help complement previous studies in understanding the extent of aflatoxin contamination in Zimbabwe to establish a basis for further investigation.

## 2. Materials and Methods

### 2.1. Sample Collection

Ten raw peanuts (500 g each) and twenty jars (375 ml) of peanut butter samples were purchased at random from different vendors at Mbare musika market, the largest informal market in Harare, the capital city of Zimbabwe. All the samples were collected during June and July 2015. All the raw peanuts and peanut butter samples were transported to Central Veterinary Laboratory in Harare, Zimbabwe for analysis. During sample collection, a structured interview was used to gather information on the vendors and peanut cottage manufacturers upon consent on where they are sourcing the peanuts, and how they were producing the peanut butter.

### 2.2. Aflatoxin Extraction and Clean up

The aflatoxin extraction was done using a QUECHERS method adapted from Frenich et al. [19]. Firstly, the raw peanut kernels were chopped up using a blender to reduce particle size and ensure sample homogeneity before subsampling. Approximately, ten grams of blended raw peanut kernels and peanut butter were weighed from each subsample into 50 ml centrifuge tubes. Then, 10 ml of acidified methanol were added into each tube and thoroughly blended using a homogeniser. The mixture was centrifuged for 12 minutes at 3500 rpm at 4°C. Then, 1.5 grams of BaSO_4_ and 0.5 grams of C18 were added into the tubes and vortexed. The vortexed mixture was centrifuged at 3500 rpm for 12 minutes at 4°C, after which the supernatant was pipetted into glass tubes and dried in a concentrator at 30°C under a stream of nitrogen gas. The residue was reconstituted through the addition of 500 *μ*l of acetonitrile and vortexed for 1 minute. The extract was filtered into HPLC vials using 0.2 *μ*m polytetrafluoroethylene membrane filters and analysed for aflatoxins.

### 2.3. Chromatography

The filtered samples and aflatoxin standards (Sigma-Aldrich, St. Louis, MO) were analysed on a liquid chromatography-mass spectrometry (LC-MS) system (Agilent 1200 infinity series) fitted with an Agilent Zorbax Eclipse C18 column (4.6 × 150 mm, 5 *μ*m pore size). The samples were injected at an injection volume of 15 *μ*l and pumped at a flow rate of 0.8 ml per minute. The mobile phase was made up of 0.1% Formic acid, Methanol, and Acetonitrile. The proportions of the mobile phase in percentage were 50%, 40%, and 10%, respectively. The Mass Spectrometry (electrospray ionisation) conditions were as follows: drying gas flow rate 9.0 l/min at a temperature of 325°C, capillary voltage of 3500 v, nebulizer pressure of 40 psi, and a fragmentor voltage of 70 v. The data was collected and analysed using the Agilent ChemStation software. Samples with toxin concentrations lower than the limit of detection (LOD) (<0.75 *μ*g/kg) were considered nondetectable. The limit of quantification (LOQ) for the samples was 1.1 *μ*g/kg. LOD was evaluated using the mean blank value + 3 × standard deviations formula, i.e, 15 blanks were analysed and the mean reading was obtained at the target retention time range. The standard deviation of those readings was also obtained. LOD = Blank + 3 × standard deviation, LOQ = Blank + 10 × standard deviation. For recovery studies during validation, 10 spiked samples were used to get an average recovery. During sample analysis, a total of 5 spiked samples were used.

## 3. Results and Discussion

**Figure 1:**
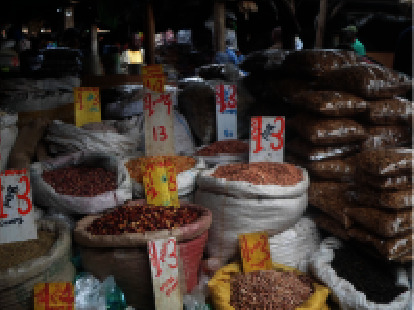
Agricultural produce on display at Mbare musika market including peanuts.

**Figure 2:**
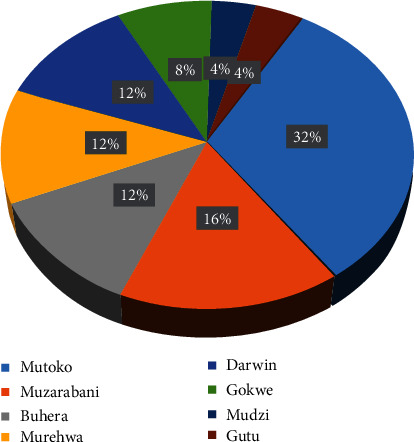
The region of source of peanuts and percentage of vendors who sourced peanuts from the region for sale at Mbare musika market.

The peanuts at Mbare musika are sold in an open market and are stored in sacks that are stacked on wooden pallets (Figure 1). The peanuts are sold mainly to household consumers, vendors, and backyard peanut butter producers. The vendors sourced most of the peanuts from Mashonaland East province with the specific areas including Mutoko (32%), Mudzi (4%), and Murehwa (12%) (Figure 2). The rest of the peanuts were sourced from Buhera (12%) in Manicaland province, Gokwe (8%) in Midlands province, Gutu (4%) in Masvingo Province, Mount Darwin (12%), and Muzarabani (16%) in Mashonaland Central. The vendors obtained their peanuts from the farmers already shelled and sorted according to grades which considered attributes such as size and colour. The peanuts which had a bigger size and were free from blemishes are considered grade A peanuts and fetch a higher price. On the other hand, shrivelled, insect infested, and splits are of low grade and they fetch a lower price. These are mainly sold to backyard peanut butter manufacturers. Generally, all the vendors at Mbare musika market did not have any knowledge of aflatoxins.

Of all the 20 peanut backyard industries sampled in this study, 16 obtained all their peanuts from the Mbare musika market. Nine out of 16 backyard manufacturers indicated that they used low grade peanuts in larger quantities for peanut butter production. The lower grade peanuts include worm and insect infested, shrivelled, split, and discoloured peanuts. The backyard industries use low grade peanuts because they were cheaper and easy to process as they did not require high temperatures during the roasting stage of peanut butter production thus lower production costs. After roasting and blanching processes, the low grade and grade A peanuts were mixed so as to produce a product with better taste as larger quantities of lower grade peanuts produce a bitter product.

The recovery for the aflatoxins at 5 *μ*g/kg spiking level for AFB_1_, AFB_2_, AFG_1_, and AFG_2_ were as follows 80.51%, 76.47%, 81.07%, and 80.78%, respectively (Table 1).

In our study, eighty percent (80%) of the raw peanut samples were contaminated with aflatoxin, with total aflatoxins ranging from 1.2 to 426.4 *μ*g/kg (Table 2). AFB_1_ was the most prevalent aflatoxin in the raw peanut samples with contamination levels ranging from 1.2 *μ*g/kg to as high as 90.8 *μ*g/kg. Aflatoxin AFB_2_ and AFG_1_ were detected in some of the samples; however, aflatoxin AFG_2_ was not detected in any of the raw peanuts. The most contaminated raw peanut sample, with a total aflatoxin level of 426.4 *μ*g/kg, contained 90.8 *μ*g/kg AFB_1_ and 335.6 *μ*g/kg AFG_1_. Aflatoxin concentrations in raw peanuts were relatively high probably due to poor storage conditions which include excessive heat, high humidity, lack of aeration and insect, and rodent damage [20, 21]. Mbare musika market is an open market characterised by poor storage conditions which could promote fungal proliferation consequently leading to high levels of aflatoxin contamination in the peanuts. The presence of aflatoxins in peanuts in Zimbabwe has been previously reported by Mupunga et al. [18]. The authors assessed fungal and aflatoxin contamination of peanuts and peanut butter in formal and informal markets in Bulawayo, the second largest city in Zimbabwe. Of the 18 peanut samples collected in their study, three (27%) were contaminated with total aflatoxins (range: 6.6-622.1 *μ*g/kg) and AFB_1_ (range: 6.3-528 *μ*g/kg). Their results indicated higher aflatoxin contamination in markets in Bulawayo compared to the findings in the current study. Furthermore, according to a study by Kamika et al. [22] which compared fungal and aflatoxin occurrence in peanuts sold at informal markets from Kinshasa, Democratic Republic of Congo (DRC) and Pretoria, South Africa, ninety percent (90%) of the samples from Pretoria indicated the presence of aflatoxins. The presence of aflatoxin in their study is of concern since 60 percent of the peanut samples in Pretoria were identified to have originated from Zimbabwe. Moreover, in another study by Maringe et al. [23], the authors found high aflatoxins in raw groundnuts grown by smallholder farmers in Makoni and Shamva districts, Zimbabwe. The findings of the current study combined with those of Mupunga et al. [18], Kamika et al. [22], and Maringe et al. [23] probably give an insight into the high prevalence of aflatoxins in raw peanuts in Zimbabwe, which is a cause for concern. In our study, AFB_2_ contamination in peanuts was in a higher concentration than the other aflatoxins in sample P5 and only AFB_2_ was present in sample P9. This pattern of aflatoxin contamination is not what is normally found in the contamination of peanuts and peanut butter. However, a similar pattern has been observed in raw groundnut in a study conducted by Maringe et al. [23] on natural postharvest aflatoxin occurrence in food legumes in the smallholder farming sector of Zimbabwe.

With regards to Zimbabwe's legislative limit for total aflatoxin in all foods, 88% of the contaminated raw peanut samples in this study exceeded the total aflatoxin level of 15 *μ*g/kg [18]. Similarly, 88% of the contaminated samples exceeded the European Union (EU) [24] limit of 4 *μ*g/kg and the Codex Alimentarius Commission of 15 *μ*g/kg for total aflatoxins [25]. Forty percent (40%) of the raw peanuts samples (4/10) exceeded the maximum limits of AFB_1_ as set by Zimbabwe legislation.

All of the peanut butter samples were contaminated with total aflatoxins ranging from 4.7 to 435.9 *μ*g/kg with a mean concentration of 98.6 *μ*g/kg (Table 3). Similar to raw peanut samples in this study, AFB_1_ was the most prevalent aflatoxin in peanut butter samples. It was detected in all of the peanut butter samples ranging from 4.7 to 382.9 *μ*g/kg with a mean concentration of 63.0 *μ*g/kg. AFG_2_ was the least prevalent aflatoxin being found only in 0.15% of the peanut butter samples. The findings from this study are similar to those of Mupunga et al. [18] who also found AFB_1_ with a high level of contamination in peanut butter samples collected in Bulawayo, Zimbabwe. In addition, the authors also found out that all the samples from the informal sector were contaminated with aflatoxins. Moreover, the authors also found high levels of aflatoxins, especially AFB_1_ in 90% (10/11) of the commercial peanut butter samples they collected. In a separate study, Njoroge et al. [26] also found aflatoxin contamination in some brands of peanut butter collected on the Zambian market but were manufactured in Zimbabwe. This could be a general indicator of the extensiveness in prevalence of aflatoxin in peanut butter products sold in Zimbabwe. This is further supported by the results from this study which indicated high levels of aflatoxins in informal peanut butter produced by peanuts from 5 provinces that supplied the Mbare informal market.

Overall, in this study, aflatoxin levels in peanut butter were higher as compared to those in raw peanuts. The high levels of aflatoxin contamination in peanut butter could be due to the use of lower grade peanuts which were poor in quality and had a higher likelihood of being infested by aflatoxins.

We compared AFB_1_ contamination in peanut butter with Zimbabwe and EU legislation. The mean concentration of 63.0 *μ*g/kg exceeded the Zimbabwe maximum limit of 5 *μ*g/kg for AFB_1_ in food by a factor >12 [18]. According to the EU maximum limit of 2 *μ*g/kg for AFB_1_ contamination in peanuts and peanut products, none of the peanut butter samples in this study complied with the legislation. Moreover, the mean concentration of 63.0 *μ*g/kg exceeded the EU maximum limit of 2 *μ*g/kg for AFB_1_ in peanuts and peanut products by a factor >31. Interestingly, 95% of peanut butter samples (19/20) exceeded the maximum limits of AFB_1_ as set by Zimbabwe legislation. A few African countries, Zimbabwe included, have adopted maximum limits for aflatoxin levels in foods meant for human consumption. However, despite the regulation being present in Zimbabwe, the results showed that aflatoxin contamination is generally high and above the maximum permissible limits in food. In developing countries, food from the informal sector rarely undergoes inspection to ensure compliance to set regulations [26, 27]. Considering the unavoidable and toxic nature of aflatoxins, since peanut butter is usually blended with cereals in infant porridge [28] and leafy green vegetable preparations [29], the results of the current study attract a public health concern. This is supported by the fact that chronic exposure to aflatoxin contamination has been linked to an increased incidence of kwashiorkor, a Protein Energy Malnutrition (PEM) which particularly occurs in infants [30, 31]. Moreover, aflatoxins have been associated with further compromising the nutritional status and immunity of AIDS victims [32, 33].

## 4. Conclusions

Aflatoxin contamination is well above the maximum allowable regulatory limits for both the local and international phytosanitary standards. Sixty percent (60%) of the raw peanut samples were contaminated with total aflatoxin ranging from <0.75 to 426.4 *μ*g/kg. Also, one hundred percent (100%) of peanut butter samples were contaminated with total aflatoxins ranging from 4.7 *μ*g/kg to 435.0 *μ*g/kg. The study shows the prevalence of aflatoxin contamination, mainly AFB_1_, in both raw peanuts and peanut butter sold at the Mbare musika market. Aflatoxin B_1_ was the most prevalent aflatoxin in both raw peanuts (range, 1.2 *μ*g/kg to 90.8 *μ*g/kg) and peanut butter (range, 4.7 to 382.9 *μ*g/kg). Forty percent (40%) of the raw peanuts and 95% of peanut butter samples exceeded the maximum limits of AFB_1_ as set by Zimbabwe legislation. Although the sample size was relatively small, the findings suggest that raw peanuts, as well as peanut butter sold at the Mbare musika market, could constitute a possible health risk to consumers, especially children who frequently consume porridge which is usually supplemented with peanut butter. Since the regulatory authorities are poorly resourced to monitor and ensure compliance, especially amongst backyard peanut butter producers, a multidimensional approach may need to be adopted. This can be achieved through coordinated efforts by health departments and related authorities to increase awareness programs for consumers, subsistence farmers, peanut vendors, and backyard peanut butter producers on the risks associated with aflatoxins. Furthermore, there is a need to promote better agricultural and storage practices amongst these stakeholders to ensure an effective reduction in dietary exposure of consumers to aflatoxin contamination.

## Figures and Tables

**Table 1:** Recoveries (%) of each aflatoxin analogue at 5 *μ*g/kg spiking level.

Aflatoxin analogue	Matrix	Recovery range %
AFB_1_	Peanut butter	80.51
AFB_2_	Peanut butter	76.47
AFG_1_	Peanut butter	81.07
AFG_2_	Peanut butter	80.78

**Table 2:** Aflatoxin contamination levels in raw peanuts sold at Mbare musika market.

Sample	AFB_1_ (*μ*g/kg)	AFB_2_ (*μ*g/kg)	AFG_1_ (*μ*g/kg)	AFG_2_ (*μ*g/kg)	Total aflatoxins (*μ*g/kg)
P1	28.6	ND	ND	ND	28.6
P2	90.8	ND	335.6	ND	426.4
P3	3.2	ND	25.0	ND	28.2
P4	16.7	ND	12.7	ND	29.6
P5	1.9	66.7	18.6	ND	87.2
P6	ND	ND	ND	ND	ND
P7	1.2	ND	ND	ND	1.2
P8	ND	ND	ND	ND	ND
P9	ND	29.1	ND	ND	29.1
P10	35.6	ND	ND	ND	35.6

Note: ND, not detected (<0.75 *μ*g/kg LOD).

**Table 3:** Aflatoxin contamination levels in peanut butter produced in Mbare backyard industry (*μ*g/kg).

Sample	AFB_1_	AFB_2_	AFG_1_	AFG_2_	Total aflatoxins	Sample	AFB_1_	AFB_2_	AFG_1_	AFG_2_	Total aflatoxins
A1	172.7	42.8	162.9	39.9	417.5	A11	11.2	ND	ND	ND	11.2
A2	98.0	20.5	94.2	74.7	287.4	A12	20.2	ND	ND	ND	20.2
A3	71.5	11.7	75.0	74.8	233.0	A13	24.0	2.7	ND	ND	26.7
A4	382.9	ND	53.1	ND	435.9	A14	40.2	ND	ND	ND	40.2
A5	148.9	ND	44.4	ND	193.3	A15	10.7	ND	ND	ND	10.7
A6	27.7	ND	ND	ND	27.7	A16	18.8	ND	ND	ND	18.8
A7	70.4	7.7	ND	ND	78.1	A17	17.6	ND	9.1	ND	26.6
A8	28.8	ND	ND	ND	28.8	A18	30.3	ND	ND	ND	30.3
A9	14.7	ND	ND	ND	14.7	A19	34.6	ND	ND	ND	34.6
A10	4.7	ND	ND	ND	4.7	A20	32.2	ND	ND	ND	32.2

Note: ND, not detected (<0.75 *μ*g/kg LOD).

## Data Availability

The data used to support the findings of this study are available from the corresponding author upon request.
